# Characteristics and outcomes of neonates hospitalised with SARS-CoV-2 infection in the UK by variant: a prospective national cohort study

**DOI:** 10.1136/archdischild-2023-326167

**Published:** 2024-04-18

**Authors:** Chris Gale, Don Sharkey, Kathryn E Fitzpatrick, Helen Mactier, Alessandra Morelli, Mariko Nakahara, Madeleine Hurd, Anna Placzek, Marian Knight, Shamez N Ladhani, Elizabeth S Draper, Cora Doherty, Maria A Quigley, Jennifer J Kurinczuk

**Affiliations:** 1 School of Public Health, Faculty of Medicine, Imperial College of Science Technology and Medicine, London, UK; 2 Academic Child Health, School of Medicine, University of Nottingham, Nottingham, UK; 3 Nuffield Department of Population Health, University of Oxford, Oxford, UK; 4 Neonatology, University of Glasgow, Glasgow, UK; 5 National Perinatal Epidemiology Unit, Oxford University, Oxford, UK; 6 UK Health Security Agency, London, UK; 7 Health Sciences, University of Leicester, Leicester, UK; 8 Neonatology, University Hospital of Wales, Cardiff, UK

**Keywords:** neonatology, COVID-19, epidemiology

## Abstract

**Objective:**

Neonatal infection with wildtype SARS-CoV-2 is rare and good outcomes predominate. We investigated neonatal outcomes using national population-level data to describe the impact of different SARS-CoV-2 variants.

**Design:**

Prospective population-based cohort study.

**Setting:**

Neonatal, paediatric and paediatric intensive care inpatient care settings in the UK.

**Patients:**

Neonates (first 28 days after birth) with confirmed SARS-CoV-2 infection who received inpatient care, March 2020 to April 2022. Neonates were identified through active national surveillance with linkage to national SARS-CoV-2 testing data, routinely recorded neonatal data, paediatric intensive care data and obstetric and perinatal mortality surveillance data.

**Outcomes:**

Presenting signs, clinical course, severe disease requiring respiratory support are presented by the dominant SARS-CoV-2 variant in circulation at the time.

**Results:**

344 neonates with SARS-CoV-2 infection received inpatient care; breakdown by dominant variant: 146 wildtype, 123 alpha, 57 delta and 18 omicron. Overall, 44.7% (153/342) neonates required respiratory support; short-term outcomes were good with 93.6% (322/344) of neonates discharged home. Eleven neonates died: seven unrelated to SARS-CoV-2 infection, four were attributed to neonatal SARS-CoV-2 infection (case fatality 4/344, 1.2% 95% CI 0.3% to 3.0%) of which three were born preterm due to maternal COVID-19. More neonates were born very preterm (23/54) and required invasive ventilation (27/57) when delta variant was predominant, and all four SARS-CoV-2-related deaths occurred in this period.

**Conclusions:**

Inpatient care for neonates with SARS-CoV-2 was uncommon. Although rare, severe neonatal illness was more common during the delta variant period, potentially reflecting more severe maternal disease and associated preterm birth.

**Trial registration number:**

ISRCTN60033461.

**WHAT IS ALREADY KNOWN ON THIS TOPIC**

- Neonatal infection with wildtype SARS-CoV-2 is rare and good outcomes predominate; the impact of later viral variants on neonates has been unclear.

**WHAT THIS STUDY ADDS**

- During the UK COVID-19 pandemic, neonatal infection with SARS-CoV-2 was rare compared with older children and adults across all viral variants; when symptomatic it was associated with respiratory problems, especially during the delta variant period when it was linked to a small number of neonatal deaths.

**HOW THIS STUDY MIGHT AFFECT RESEARCH, PRACTICE OR POLICY**

- Rapidly established and ongoing national surveillance was essential to understand the neonatal impact of the evolving SARS-CoV-2 pandemic, highlighting the key role of established systems in future pandemics.
- The long-term effects of early life exposure to SARS-CoV-2 are unknown, and ongoing data collection, linkage and developmental follow-up remain crucial.

## Introduction

Children are less severely affected by SARS-CoV-2 than adults, and this pattern has been seen across viral variants.^1^ In the neonatal period (first 28 days), neonates can be infected by SARS-CoV-2 or indirectly affected because of maternal infection, for example, through spontaneous or iatrogenic preterm birth. We have previously reported that neonates were more likely than older children to require respiratory support following SARS-CoV-2 infection when the wildtype variant was dominant,^2^ but it is not clear if this pattern persisted in later periods. The alpha and delta variants of SARS-CoV-2 resulted in more severe maternal SARS-CoV-2 infection,^3^ but there are few studies describing the impact of SARS-CoV-2 viral variants in neonates.^4^

Using population-level active surveillance data linked to maternal, neonatal and paediatric intensive care and perinatal mortality data, we aimed to describe the epidemiology, clinical course and short-term outcomes of neonates with confirmed SARS-CoV-2 infection cared for in hospitals in the UK over the first 2 years of the pandemic, stratified by the dominant SARS-CoV-2 variant in circulation at the time of infection.

## Methods

This was a national prospective cohort study using the British Paediatric Surveillance Unit (BPSU).^5^ From 1 April 2020, senior paediatricians (~4000) in all 155 hospital trusts and health boards in the UK with their associated 190 neonatal units (NNUs) received a weekly (until March 2021) then monthly electronic BPSU reporting card asking them to report any baby who had laboratory-confirmed SARS-CoV-2 infection in the first 28 days after birth and received inpatient care on a postnatal ward, NNU, paediatric inpatient ward or paediatric intensive care unit (PICU). Well neonates born in the UK remained with their mother on postnatal wards until mother and baby were fit for discharge; neonates with asymptomatic SARS-CoV-2 on postnatal wards and asymptomatic cases detected through screening were not reported. Monthly reporting cards sought confirmation that all eligible neonates in the previous month had been reported, and that any reports of no infected neonates were accurate (active negative surveillance). To maximise case ascertainment, we linked to national testing data from Public Health England and Health Protection Scotland between 1 March 2020 and 31 March 2021, as well as the Paediatric Intensive Care Audit Network,^6^ United Kingdom Obstetric Surveillance System (UKOSS) data^7^ and MBRRACE-UK national perinatal mortality surveillance data.^8^ Additional details can be found in the online supplemental methods. Intensive care was defined using British Association of Perinatal Medicine categories of care^9^ for neonatal admissions, or any admission to a PICU. Severe disease was defined as having received respiratory support. Very preterm birth was defined as birth at <32 gestational weeks.

10.1136/fetalneonatal-2023-326167.supp1 Supplementary data

Neonatal deaths were verified with the MBRRACE-UK national surveillance of perinatal deaths.^10^ Neonatal deaths were attributed to SARS-CoV-2 if the treating paediatrician reported that SARS-CoV-2 infection contributed to the baby’s death; we also recorded if the referring clinician reported that maternal SARS-CoV-2 infection led to spontaneous or iatrogenic preterm birth.

UK SARS-CoV-2 testing policy among pregnant women and neonates evolved during the study. Initially, only symptomatic women and neonates were tested. Routine screening of all obstetric admissions was recommended by the Royal College of Obstetricians and Gynaecologists on 29 May 2020 and neonatal testing recommended for symptomatic neonates of mothers with a SARS-CoV-2 infection; testing of asymptomatic neonates varied. Confirmation of neonatal SARS-CoV-2 infection required at least two positive samples, including one at least 72 hours after birth.^11^ UK policy was that well neonates of SARS-CoV-2-infected mothers should be cared for alongside their mother in the postnatal ward and not routinely tested.

This analysis presents characteristics and outcomes for neonates reported as having confirmed SARS-CoV-2 infection in the first 28 days after birth, between 1 March 2020 and 1 April 2022, and for whom complete data had been received by 30 July 2022. To provide a complete description of the first two pandemic years, this report includes neonates with SARS-CoV-2 infection between 1 March 2020 and 30 April 2020 previously reported.^2^

As individual-level SARS-CoV-2 variant data were not recorded in medical records, the outcomes were compared across four proxy groups according to the period in which the original wildtype, alpha variant, delta variant or omicron variant was the dominant circulating strain in the UK.

The original wildtype period included neonates diagnosed from 1 March to 30 November 2020, the alpha variant period from 1 December 2020 to 15 May 2021, the delta variant period from 16 May 2021 to 14 December 2021 and the omicron variant period from 15 December 2021 to 1 April 2022. We chose cut-off dates for the delta and omicron periods using data on variant sequencing from Public Health England to identify the week when these variants first contributed >50% of SARS-CoV-2 infections nationally.^12^ Since genomic data on the variant were less widely available at the start of the pandemic, Public Health England modelled proxy data and reported that the alpha variant reflected the substantial majority of infections across all areas of England during December 2020; therefore, 1 December 2020 was used as an estimated cut-off date.^13^

### Parent, patient and public involvement

Parents, patients and the public were consulted during the design of the study and presentation of the findings through the MBRRACE-UK third-sector stakeholder group and the NIHR Policy Research Unit in the Maternal and Neonatal Care Public and Patient Involvement group.

### Statistical analysis

Descriptive statistics are presented as frequencies, proportions and medians with IQRs, as appropriate.

## Results

**Figure 1:**
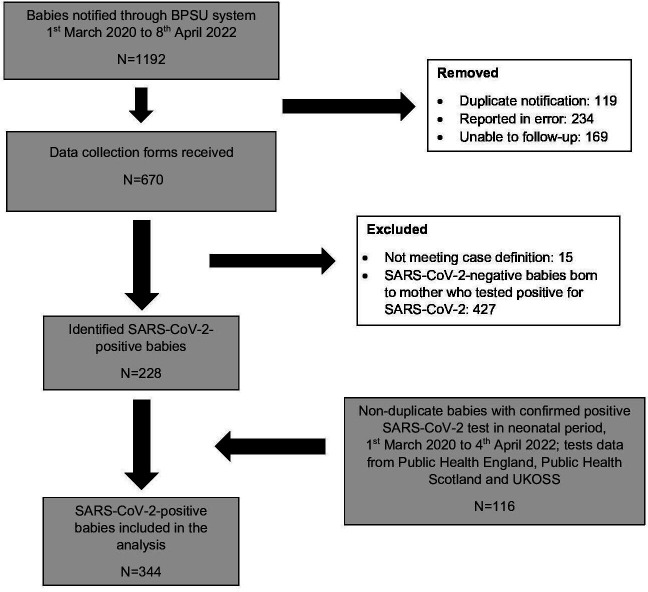
Flow chart of case selection for study period 1 March 2020 to 1 April 2022. BPSU, British Paediatric Surveillance Unit; UKOSS, United Kingdom Obstetric Surveillance System.

**Figure 2:**
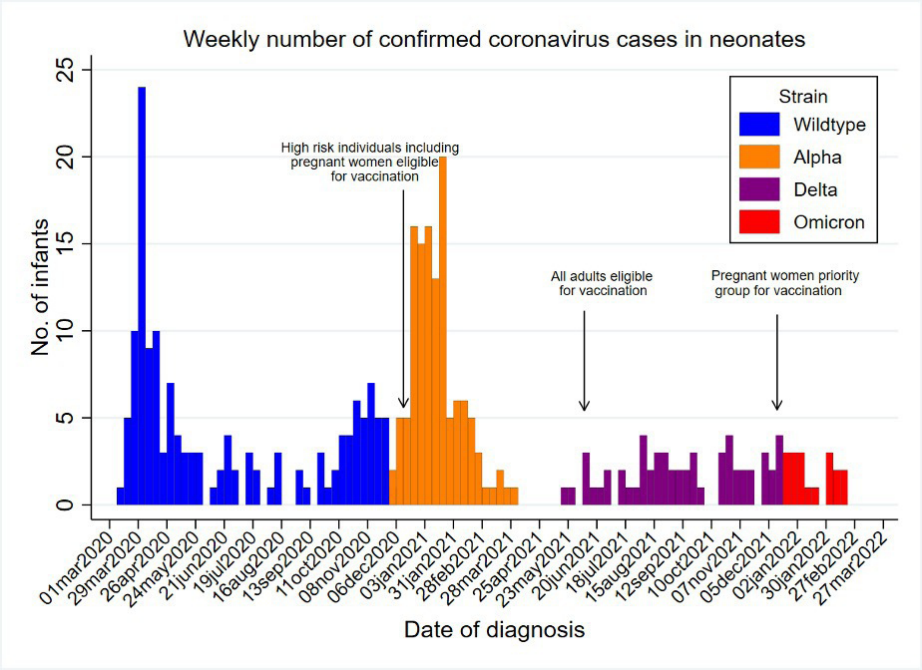
Weekly confirmed neonatal SARS-CoV-2 infections by dominant circulating variant in the UK.

Monthly BPSU card returns were received between 91.3% (3748/4070, April 2020) and 71.9% (3110/4298, February 2022) of UK paediatricians over the surveillance period. In total, 1192 potentially eligible neonates were reported to the BPSU system over the study period and 116 non-duplicate neonates were identified from other sources (figure 1). Linkage with data held in the NNRD was achieved for 99% (132/134) of neonates reported through the BPSU system who received NNU care. Three hundred and forty-four neonates were diagnosed with SARS-CoV-2 infection in the first 28 days after birth and received inpatient care, predominantly in the wildtype-dominant and alpha-dominant periods, with numbers dropping in subsequent variant periods: 146 neonates were reported in the wildtype period, 123 in the alpha period, 57 in the delta and 18 in the omicron periods (figure 2). Three hundred and twenty cases were in England, 16 cases in Scotland, 6 in Wales or Northern Ireland; country data were missing for 2 cases.

Median age at diagnosis was 9 days (IQR 3–17 days); 44.5% (153/344) of the neonates were diagnosed in the first 7 days after birth; of these, 66.7% (102/153) were born to a mother with confirmed SARS-CoV-2 infection within 7 days before or after giving birth. The age distribution at diagnosis was similar across different dominant circulating variant periods (online supplemental figure 1). Respiratory signs were common along with poor feeding or vomiting and fever (online supplemental figure 2); 15% of neonates were asymptomatic.

Neonates in hospital with neonatal SARS-CoV-2 infection were more commonly preterm and male when compared with all live births in England and Wales and this was consistent across all variant epochs, although sex differences were less marked in the alpha period (table 1). The highest proportions of very preterm births were seen in the delta-predominant period. An over-representation of non-white ethnic groups among neonatal SARS-CoV-2 infections in the wildtype period was not seen with later variants (table 1).

**Table 1:** Background characteristics of neonates in hospital with SARS-CoV-2 infection by dominant circulating variant; distribution of background characteristics in all UK live births in 2021^14^

	Distribution in live births in England and Wales 2021 (%)	All variants N (%)*	Wildtype N (%)*	Alpha N (%)*	Delta N (%)*	Omicron N (%)*
Total cases		344	146	123	57	18
Gestation at birth						
<28^+0^	0.5	11 (3.3)	5 (3.6)	2 (1.6)	3 (5.6)	1 (5.6)
28^+0^–31^+6^	0.8	41 (12.3)	8 (5.7)	12 (9.8)	20 (37.0)	1 (5.6)
32^+0^–36^+6^	6.3	79 (23.7)	27 (19.3)	34 (27.9)	12 (22.2)	6 (33.3)
≥37	92.1	203 (60.8)	100 (71.4)	74 (60.7)	19 (35.2)	10 (55.6)
Missing	–	10	6	1	3	0
Sex						
Male	51.2	188 (55.0)	84 (57.9)	63 (51.2)	31 (55.4)	10 (55.6)
Female	48.8	154 (45.0)	61 (42.1)	60 (48.8)	25 (44.6)	8 (44.4)
Missing	–	2	1	0	1	0
Ethnicity						
White	70.5	230 (70.3)	89 (61.8)	80 (71.4)	46 (86.8)	15 (83.3)
Asian/Asian British	12.2	60 (18.3)	34 (23.6)	19 (17.0)	5 (9.4)	2 (11.1)
Black/African/Caribbean/Black British	4.8	19 (5.8)	11 (7.6)	6 (5.4)	1 (1.9)	1 (5.6)
Mixed/Other	9.1	18 (5.5)	10 (7.0)	7 (6.3)	1 (1.9)	0 (0)
Missing	–	17	2	11	4	0

*Percentage of those with complete data; duration of variant-predominant periods were not equal.

**Figure 3:**
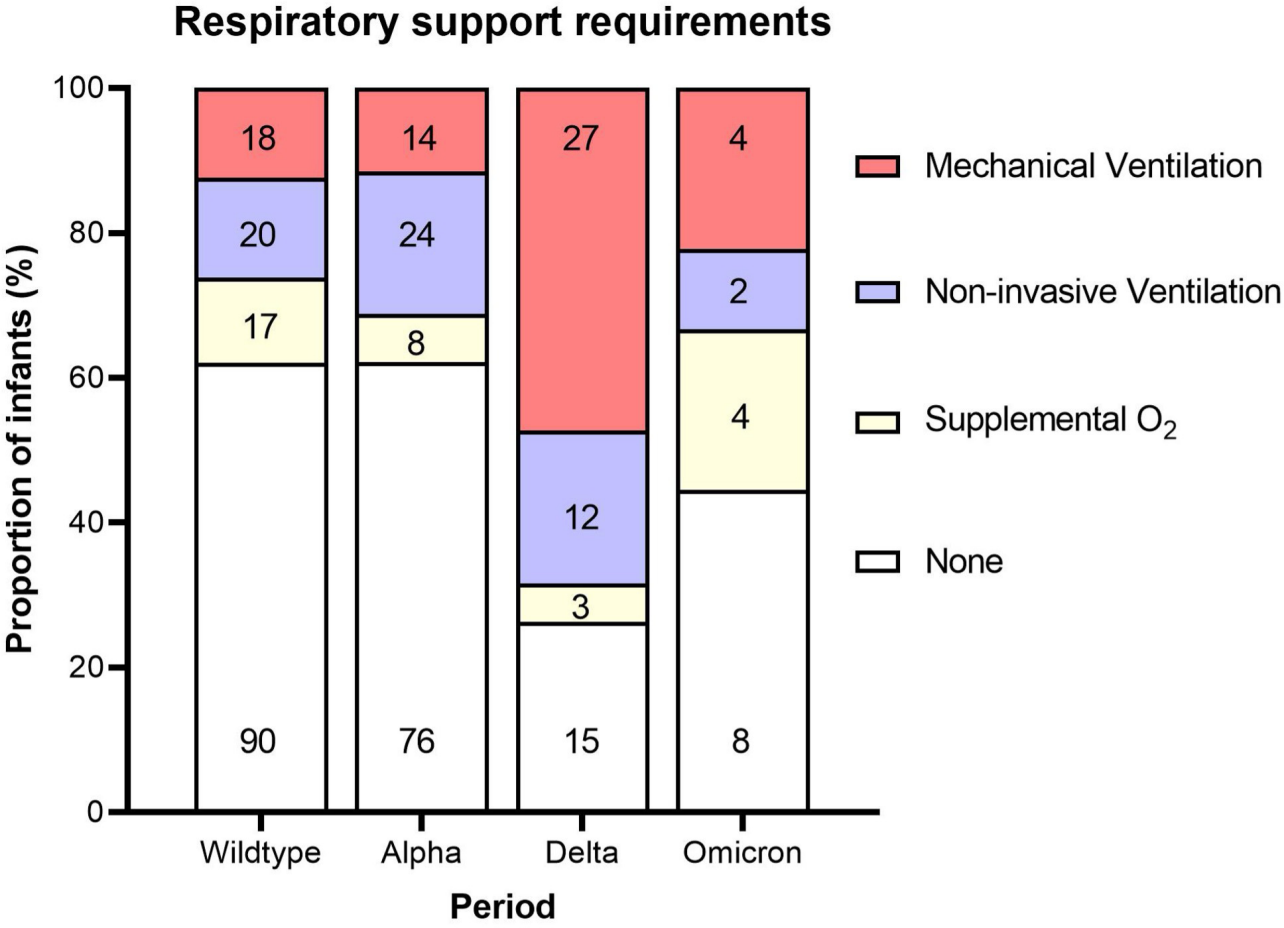
Maximum respiratory support requirements of hospitalised neonates with SARS-CoV-2 infection during the dominant circulating variant period (wildtype n=145, alpha n=122, delta n=57 and omicron n=18).

Most neonates in hospital with neonatal SARS-CoV-2 infection did not require high-level care; however, 44.7% required some form of respiratory support and 21.8% received intensive care (table 2). Higher numbers and proportions of neonates in hospital with neonatal SARS-CoV-2 in the delta period required intensive care or invasive respiratory support, compared with other variant periods (figure 3). Infection following suspected nosocomial transmission, excluding vertical transmission, affected 7.8% of neonates overall. Outcomes following neonatal SARS-CoV-2 infection were generally good; however, 11 deaths occurred in neonates who tested positive for SARS-CoV-2, 4 of which were attributed either directly or indirectly to neonatal SARS-CoV-2 infection (case fatality 4/344, 1.2% 95% CI 0.3% to 3.0%). Three of these four neonates had been born preterm due to maternal COVID-19. All four SARS-CoV-2-related neonatal deaths were in the delta period.

**Table 2:** Clinical care received and outcomes following neonatal SARS-CoV-2 infection during the dominant circulating variant periods

	All variants N (%)*	Wildtype N (%)*	Alpha N (%)*	Delta N (%)*	Omicron N (%)*
Total cases	344	146	123	57	18
Transmission					
Maternal SARS-CoV-2 infection at birth	133 (38.7)	45 (30.8)	54 (43.9)	25 (43.9)	9 (50.0)
Suspected nosocomial transmission	27 (7.8)	18 (12.3)	3 (2.4)	4 (7.0)	2 (11.1)
Highest level of care					
Intensive care	75 (21.8)	23 (15.8)	18 (14.6)	28 (49.1)	6 (33.3)
Non-intensive care	269 (78.2)	123 (84.2)	105 (85.4)	29 (50.9)	12 (66.7)
Required respiratory support					
Yes	153 (44.7)	55 (37.9)	46 (37.7)	42 (73.7)	10 (55.6)
No	189 (55.3)	90 (62.1)	76 (62.3)	15 (26.3)	8 (44.4)
Missing	2	1	1	0	0
Neonatal outcome					
Discharged home	322 (93.6)	137 (93.8)	119 (96.8)	50 (87.7)	16 (88.9)
Transferred to another site/still admitted	11 (3.2)	7 (4.8)	3 (2.4)	1 (1.8)	0 (0)
Died	11 (3.2)	2 (1.4)	1 (0.8)	6 (10.5)	2 (11.1)
Death related to SARS-CoV-2	4	0	0	4	0

*Percentage of those with complete data.

As expected in any neonatal population, degree of prematurity was strongly linked with receipt of respiratory support with all extremely preterm neonates infected with SARS-CoV-2 requiring ventilation; this highlights the challenges separating severe manifestations of SARS-CoV-2 infection from conditions related to preterm birth (online supplemental table 1).

## Discussion

Using population-level active surveillance data from March 2020 to April 2022 and spanning wildtype, alpha, delta and omicron variant-predominant periods during which there were approximately 1.4 million births recorded in the UK,^14–16^ we confirm that the need for inpatient care for neonates with SARS-CoV-2 infection was rare and outcomes were generally good. Neonatal SARS-CoV-2 infection led to severe disease in a minority of neonates, with death related to neonatal SARS-CoV-2 infection occurring in 1.2% of hospitalised neonates. During the delta variant-predominant period, higher numbers of neonates had severe disease associated with SARS-CoV-2 infection, defined as requiring respiratory support, compared with other variant epochs. We believe this is the first study to present descriptive data from a national cohort on neonatal deaths reported as related to SARS-CoV-2 infection, all of which occurred in the delta-predominant period in the UK.

The pattern of more serious neonatal disease in the delta period described here may be explained by the higher number and proportion of neonates born very preterm with neonatal SARS-CoV-2 infection during this period. More severe maternal infection and higher rates of preterm birth have been described in the delta-predominant wave in the UK^3^ and internationally^17^; it is unclear whether this pattern of more severe maternal disease was related to changing characteristics of the viral variant or changes in behaviour and low vaccination rates among pregnant women in the UK during this period. Similarly, it is not possible to determine from these data presented whether the delta variant was more pathogenic in neonates, or whether adverse neonatal outcomes were a result of more severe maternal SARS-CoV-2 disease leading to more preterm births, a well-described risk factor for neonatal infection.^18^

There have been very few population-based studies of neonates infected with SARS-CoV-2 infection, and data describing neonatal SARS-CoV-2 infection by variant are even more sparse. Population-level data from Germany describing paediatric SARS-CoV-2 infection show higher rates of COVID-19-related hospitalisation and intensive care unit admission for children under 5 years in the delta-predominant period compared with alpha-predominant, wildtype-predominant and omicron-predominant periods,^19^ although data from the UK did not report a higher rate of intensive care admissions for children under 5 with delta compared with earlier viral variants.^20^ Data covering approximately 10% of the US population found similar rates of intensive care admissions of infants <6 months of age with the delta and omicron variants.^21^ A further study examining administrative data representing approximately 20% of US hospital admissions found that COVID-19 among newborns is rare but is associated with newborn critical care outcomes like invasive ventilation, and that risks for invasive compared with non-invasive ventilatory support were higher in delta compared with pre-delta periods,^22^ consistent with the UK data we present here. Single-centre case-series have also reported more severe SARS-CoV-2 infection in infants and neonates in delta-predominant compared with other variant-predominant periods,^4^ supporting a link between infection with the delta variant and more severe neonatal disease.

Although there have been multiple registries describing neonatal SARS-CoV-2 infection,^23 24^ there have been very few representative population-level studies and those that have published have been limited by low case numbers.^25^ The population-level data we present here include and build on previously published neonatal surveillance data from the UK from the first weeks of the SARS-CoV-2 pandemic when the wildtype variant predominated.^2^ These updated data describe the presentation and clinical course of neonatal SARS-CoV-2 infection in the largest number of neonates reported to date. Consistent with early data,^2^ we confirm that while respiratory signs were widespread, symptomatic neonatal SARS-CoV-2 most commonly presented with poor feeding or other gastrointestinal signs. Fever and respiratory signs were also common but did not predominate as in other paediatric groups.^26^ Data from Switzerland in one of the few other population-level surveillance studies found fever and respiratory signs most common at presentation in the 73 neonates reported,^25^ possibly reflecting the non-specific nature of these neonatal signs. The large number of neonatal cases we report compared with other neonatal studies provide confidence in our key findings that while receipt of respiratory support is relatively common among neonates hospitalised with neonatal SARS-CoV-2, this is generally seen in the context of preterm birth. Almost half of hospitalised neonates with SARS-CoV-2 infection received some form of respiratory support. We also report reassuring short-term outcomes following neonatal SARS-CoV-2 infection consistent with Swiss national data^25^ and international registry data.^27 28^

The high rate of presumed nosocomial transmission reported throughout the study (7.8%) is of concern. This likely reflects the challenges of limiting viral exposure in NNUs with few isolation facilities, in the context of a pandemic with high rates of staff and parent infection.

### Strengths and limitations

A key strength of this national prospective cohort study was the use of an established active surveillance system with high reporting rates by UK paediatricians throughout the study period. The long-standing monthly BPSU reporting cards were augmented by additional weekly reporting and supplemented by national virology testing during the first year. Data were also linked to national obstetric surveillance data, paediatric intensive care national audit data, routinely recorded neonatal data and national perinatal mortality surveillance throughout the study period to ensure comprehensive case ascertainment and nationally representative disease severity and outcome data. Use of such established national reporting systems minimises selection bias. By limiting the study to neonates in hospital with SARS-CoV-2 infection, we focused on the more severe spectrum of disease, which is of most interest to health services and clinicians. The main limitation of this approach is that neonates with less severe SARS-CoV-2 in the community were not included and hence true population incidence and asymptomatic infection rates are not possible to quantify. Linked population-level data from Scotland which included community testing data found that two in three neonates with SARS-CoV-2 infection received hospital care,^29^ suggesting that the true incidence of neonatal infection in the whole UK including community testing was around 500–550 neonates—still rare compared with older children and adults. Other limitations of this study include the challenges of separating out the effects of SARS-CoV-2 infection per se from other common causes of neonatal illness, primarily preterm birth which commonly requires respiratory support, and the lack of an agreed severity definition for neonatal infection. We did not have access to viral variant sequencing data for individual neonates and hence we used proxy time periods to ascribe variants. In addition, national guidance for neonatal testing changed over the study period, particularly in the early stages of the pandemic. This may have led to underestimation of the actual numbers of SARS-CoV-2 infection during the wildtype-dominant period. The lower number of BPSU reporting card returns in the omicron-dominant period may reflect reporting fatigue by this point in the pandemic, and may thus also underestimate the number of mildly affected neonates admitted during this period.

## Conclusions

Inpatient care for neonates infected with SARS-CoV-2 was uncommon throughout the first 2 years of the pandemic and short-term outcomes were generally good. Severe disease was more common, although still rare, during the delta variant period; this may have been influenced by more severe maternal disease resulting in more very preterm neonates. Rapidly established and ongoing national surveillance was essential to understand the neonatal impact of the evolving SARS-CoV-2 pandemic, highlighting the key role of established systems such as the BPSU, UKOSS and MBRRACE-UK perinatal mortality surveillance. The long-term effects of early life exposure to SARS-CoV-2 are unknown, and ongoing data collection, linkage and developmental follow-up remain crucial.

## Data Availability

Data are available on reasonable request. The datasets generated during and analysed during the current study are available from the corresponding author on reasonable request.
